# Barriers to accessing TB diagnosis for rural-to-urban migrants with chronic cough in Chongqing, China: A mixed methods study

**DOI:** 10.1186/1472-6963-8-202

**Published:** 2008-10-02

**Authors:** Qian Long, Ying Li, Yang Wang, Yong Yue, Cheng Tang, Shenglan Tang, S Bertel Squire, Rachel Tolhurst

**Affiliations:** 1 School of Public Health, Chongqing Medical University, No.1 Yixueyuan Road, Chongqing, PR China; 2 College of Military Preventive Medicine, Third Military Medical University, PLA, No. 30 Gaotanyan Road, Shapingba District, Chongqing, PR China; 3 Chengdu Municipal Center for Disease Control and Prevention, No.4 Longxiang Road, Chengdu, PR China; 4 Jiulongpo District Center for Disease Control and Prevention, No.56 Panlong Road, Chongqing, PR China; 5 Liverpool School of Tropical Medicine, Pembroke Place, Liverpool, L3 5QA, UK

## Abstract

**Background:**

China is facing a significant tuberculosis epidemic among rural-to-urban migrants, which poses a threat to TB control. This study aimed to understand the health seeking behaviour of and health systems responses to migrants and permanent urban residents suffering from chronic cough, in order to identify the factors influencing delays for both groups in receiving a TB diagnosis in urban China.

**Methods:**

Combining a prospective cohort study of adult suspect TB patients and a qualitative study, the Piot model was used to analyze the health seeking behaviour of TB suspects among migrants and permanent urban residents, the factors influencing their decision and the responses by general health providers. Methods included a patient survey, focus group discussions with migrants in the general population, qualitative interviews with migrant and permanent resident TB suspects and TB patients as well as key stakeholders related to TB control and the management of migrants.

**Results:**

Sixty eight percent of migrants delayed for more than two weeks before seeking care for symptoms suggestive of TB, compared to 54% of residents (p < 0.01). When they first decided to seek professional care, migrants were 1.5 times more likely than residents to use less expensive, community-level health services. Only 5% were ultimately referred to a TB dispensary. Major reasons for both patient and provider delay included lack of knowledge and mistrust of the TB control programme, lack of knowledge about TB (patients), and profit-seeking behaviour (providers). In the follow up survey, 61% of the migrants and 41% of the residents who still had symptoms gave up continuing to seek professional care, with a statistically significant difference between the two groups (p < 0.05).

**Conclusion:**

Rural-to-urban migrants are more likely than permanent residents to delay in seeking care for symptoms suggestive of TB in urban Chongqing. 'Patient-' and 'provider-' related factors interact to pose barriers to TB diagnosis for migrants, including: low awareness, and poor knowledge among both the general public and TB suspects about TB as a disease and about the TB control programme; low financial capacity to pay for care and diagnostic tests; and inadequate use of diagnostic tests and referral to TB dispensaries by general health providers.

## Background

International mass migration is increasingly being identified as a significant factor in the international resurgence of tuberculosis [1] whilst poverty and social exclusion continue to shape the epidemiology of the disease and pose challenges to its control [2]. China has the second largest population of TB patients in the world, of whom 80% live in rural areas [3]. After two decades of comprehensive socio-economic reforms, a significant change in Chinese society is the rising population mobility, particularly the movement from rural to urban areas due to rural-urban income disparity and rural labour surplus [4]. This has been identified as a major challenge facing TB control [5]. Available data show that whilst the incidence of pulmonary TB among permanent urban residents in major Chinese cities is relatively stable [6,7], incidence amongst rural-to-urban migrants is increasing annually. The number of active TB patients in this group has increased by 9.4% every year between 1997 and 2001.

Poverty and social exclusion are likely to fuel the epidemic amongst migrants. In moving from the rural to urban context, migrants are more vulnerable to TB infection due to their relatively poor living and working conditions [8]. The relative difficulty of obtaining permission to live and work in cities often leads to their exclusion from full citizenship and welfare structures. More than a dozen certificates and approvals are required, to be renewed annually, with the process taking at least three months, and costing between 500 and 1000 Chinese Yuan (about one month's salary). If they fail to obtain this permission, many migrants choose to live and work in cities illegally, which restricts their access to formal employment, secure housing, local community services including healthcare and health insurance, the rural Cooperative Medical System and the urban health insurance and social welfare schemes [9,10]. In the absence of health insurance, migrants have to pay for healthcare services out-of-pocket, and face higher costs of care than those in the rural areas. Weakened family support and social networks also reduce access to care [9].

The majority of TB cases in China are detected by passive case finding. Although free TB diagnosis and treatment is in theory available at designated TB dispensaries which are usually one of the departments of the Center for Disease Control and Prevention (CDC), heavy costs are often incurred in the process of health seeking and (non)referral from general health facilities [11]. The range of difficulties facing migrants in accessing TB diagnosis suggests that case detection is likely to be lower amongst this group than permanent urban residents, which constitutes both a rights issue and a threat to public health.

It is also hypothesised that migrants may face longer delays in obtaining a TB diagnosis than urban residents. International studies have tended to distinguish between delays by patients in seeking initial care (known as 'patient' or 'personal' delay) and delays by the health system in reaching a diagnosis once the patient enters the system (known as 'provider' or 'system' delay). In China, most studies on the issue of delay focus on the rural areas. A study in rural Shandong reported that the median total delay from the onset of symptoms to TB diagnosis was 57 days. Patients with a longer patient delay experienced a shorter health system delay [12]. However, a study in the rural areas of four provinces (Fujian, Xinjiang, Inner Mongolia and Liaoling) in 2002 identified serious provider delay, with 30–60% of patients experiencing a delay in receiving a TB diagnosis after first seeking care [13]. Little is known about either 'patient' or 'provider' delays for rural-to-urban migrants. The study reported in this paper aims to understand the health seeking behaviour of migrants for symptoms suggestive of TB, and to identify the factors influencing delays in receiving a TB diagnosis for this group in urban China. Mindful of the false dichotomy between 'patient' and 'provider' delay [12], we have used the Piot model to produce an integrated analysis of diagnostic delays faced by patients.

## Methods

The study combined both quantitative and qualitative methods to capture both the extent and a holistic understanding of the factors affecting delay.

### Study setting

Chongqing Municipality is located in western China, and includes both rural and urban areas. The Municipality hosts large numbers of rural-to-urban migrants and has a high TB prevalence rate. According to the Fourth National TB Survey in 2000, the TB prevalence rate for Chongqing Municipality (including urban and rural areas) was 112.5 per 100,000. The smear positive TB prevalence rate was much higher in rural counties of Chongqing (173.0/100,000) than that in the urban areas (81.1/100,000). Currently, there is no available data to estimate the TB prevalence among rural-to-urban migrants in the study areas.

Two districts of Chongqing city, the central Yuzhong district (YZ) and the suburban Jiulongpo district (JLP), were selected on the basis that they have around 40% of migrant workers in their population and represent different characteristics of Chongqing in terms of economic, social and health service development. In 2002, YZ and JLP had per capita average income at 13,426CNY and 5822CNY and 496 and 381 health facilities, respectively.

### Quantitative study

#### Study subjects

A prospective cohort study of adult TB 'suspects' (over 15 years old) experiencing chronic cough for more than three weeks or sputum with blood or hemoptysis was carried out over a period of 3 months in 23 randomly selected general health facilities in the two districts, covering tertiary, secondary and primary health facilities. The TB suspects in migrants and permanent urban residents (we will refer to them as 'residents' in this paper) were recruited in the patient survey in a proportion of 1:4, which reflects the proportion of the migrants among the whole population in the urban areas of Chongqing (an estimated 24% in Chongqing city in 2004) [14].

#### Data collection

Two standardised questionnaires were used to collect information on the recruited TB 'suspects' health care seeking experiences. Questionnaire (A) covered subjects' general demographic and socio-economic information, their knowledge of TB, and their health care seeking experiences from the onset of the current symptoms to the interview, including self care, the pattern of health facilities selected, and diagnostic tests received. This was not necessarily the first care-seeking visit of subjects since the onset of symptoms. The study subjects were followed up after a three-month interval, using Questionnaire (B) to find out their pathway to seeking care (including the types of health facilities used, referral, and diagnostic tests) and health outcomes over the follow up period.

### Qualitative study

#### Study population

Focus Group Discussions (FGD) were held with 12 groups of women and men in the general rural-to-urban migrant population. Semi-structured individual interviews were conducted with: 20 TB 'suspects' recruited by the above survey; 17 newly registered TB patients who were identified to have experienced delays in receiving a TB diagnosis; and 23 key informants related to TB control, including health managers and health workers from the Center for Disease Control and Prevention (CDC) and general health facilities at different levels; and community organizations that have contact with the migrants.

#### Data collection

Focus group discussions with migrants in the general population explored use and experiences of healthcare, knowledge and perceptions about TB and the TB control programme. Interviews with TB suspects and patients investigated their pathway to care and the factors affecting their health seeking behaviour. Key informants were asked about their perceptions of the challenges facing TB case identification with a focus on the particular issues with regard to migrants.

### Data Analysis

The Piot model is an operational analysis model that has proven useful for analyzing case management (passive case finding and treatment) in disease control programmes such as tuberculosis, malaria and sexually transmitted infections [15,16]. In the case of TB control the model describes the different steps that individuals in the community go through after becoming ill with symptoms related to TB until finally being cured by the TB control programme. The main 'steps' or aspects for analysis include incidence in the programme area, patients' awareness of TB, their motivation to seek care at a health care delivery point, selection of TB suspects by a health professional, appropriate examination carried out, sensitivity of the examination, correct treatment, regular treatment taken by the TB patient and effectiveness of the treatment [17].

We utilized the Piot model as the analytical framework for this paper, with some modifications for a focus specifically on case finding. The main steps are summarized in Table 1.

**Table 1:** Framework of problem analysis for TB case finding

Step 1	Awareness --	The patient is aware that he/she is suffering from symptoms or complaints, which could be TB	
Step 2	Motivation --	The patient suffering from symptoms which could be TB contacts a health care delivery point	Case
Step 3	Selection --	The health professional suspects TB and requests a X-ray and sputum examination (smear)	Finding
Step 4	Examination --	The sputum test is correctly carried out on the patients thus selected	

Student's *t*-test and χ^2 ^test were conducted to compare the difference between the two populations studied (residents and migrants) and mean, median and proportion were utilized in descriptive analysis of the quantitative data. The population was divided into three income groups with reference to the 2004 local (Chongqing) poverty line (2340 yuan/year) [18] for analysis: those living on less than 2 times the poverty line, 2–3 times the poverty line and over 3 times the poverty line.

With the permission of each participant, interviews and FGDs were taped and transcribed into standard Chinese. The resulting qualitative data were analysed using the 'framework approach', whereby a framework was constructed using the topic guide, checklists and categories emerging from the transcripts, and applied to the data to identify themes [19].

### Quality assurance

In the patient survey, 10% of subjects were re-interviewed for quality control and about 95% of the results were almost identical on double checking. The trustworthiness of qualitative data was assured by triangulating findings from different respondents and methods, and extensive consultation, cross-checking of results and analysis with stakeholders.

### Ethical considerations

This study obtained the approval of the WHO Scientific Committee for Research in Human Subjects (SCRIHS), the Research Ethics Committee at the Liverpool School of Tropical Medicine and the University President's Committee at Chongqing Medical University. All of the data were collected with the informed consent of participants prior to their participation in the study.

### Study limitations

Our study methodology has a number of limitations. First, the study focuses on suspected TB cases and we are not able to determine the true prevalence of TB amongst this population due to the difficulties in following up a proportion of cases (32% of residents and 34% of migrants were not re-interviewed). We are therefore unable to assess the extent, severity and reasons for delays in diagnosis solely for those actually suffering from TB, and some delays reported may be due to 'suspects' not in fact having the disease. However, the methodology does identify the factors influencing the ability of TB suspects (including TB patients) to enter and progress through the health system to achieve a diagnosis (positive or negative), which has important implications for improving the ability of the system to increase the case detection rate. Second, attempts were made to contact all patients for a second interview, but it was not possible to reach all of them because of population mobility and incorrect contact information, and some patients refused a further interview. Therefore there may have been a selection bias in the sample for the follow-up survey because of the significant numbers of original interviewees that we were unable to follow up. The results of this survey should therefore be interpreted with caution. However, they do identify continuing barriers to diagnosis for some patients.

## Results

### Socioeconomic and demographic characteristics of study subjects

**Figure 1:**
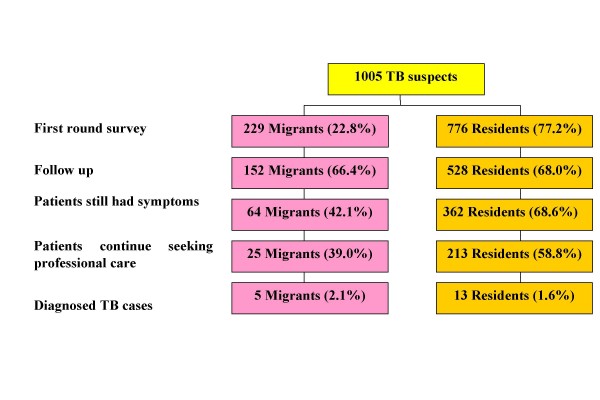
**Progress in diagnosis by TB suspects**. The flow chart shows the progress in diagnosis by TB suspects among migrants and residents over the time of the two surveys conducted. In the following up, only 39% of the migrants who were still suffering from the symptoms continued seeking professional care, compared to 59% of the residents.

A total of 1005 TB suspects, of whom 776 were residents and 229 were migrants, were recruited in the first questionnaire survey. Figure 1 provides general information on the number of patients involved in the two surveys in the two study groups and their basic progress towards diagnosis at these two points in time. Three months after the first survey, 680 interviewees (528 residents (68%) and 152 migrants (66%)) were successfully followed up. Among them, 426 interviewees (362 residents (69%) and 64 migrants (42%)) still had symptoms – 94% of patients had a cough and 6% of them had haemoptysis (sputum with blood) – but only 238 patients (56%) sought professional care. Only 39% of the migrants who were still suffering from symptoms continued seeking professional care, as compared to 59% of the residents. By the end of the follow-up study, 18 TB cases (13 residents and 5 migrants), were diagnosed.

About 70% of the migrants were aged 15 to 44, while more than half of the residents were over 60 years old. Forty four percent of the migrants had an educational level of elementary school or below, compared to 37% of the residents. A higher proportion (63 %) of migrants than residents (47 %) were in the lowest income group (under 2 times the poverty line). In addition, 94% of the migrants were not covered by any health insurance, three times higher than the proportion of the residents studied.

The general migrants, TB suspects and TB patients sampled in the qualitative study included both women and men and a wide age range from 18 to over 60 years old. Most of the recruited migrants had elementary school level education, and worked on a part-time or temporary basis in enterprises or factories or were self-employed.

The following sections present the findings from both the quantitative and qualitative studies using the Piot model as an analytical framework.

### Knowledge and awareness of TB and the TB control programme

Knowledge and awareness of TB among the general population (migrants) and TB suspects was influential on their health seeking behaviour. The survey found that TB suspects, and especially the migrants, had low levels of knowledge and awareness of TB. As Table 2 shows, residents had better knowledge about TB than migrants, and the difference is statistically significant in questions 2,3,4,5 (p < 0.01). Most of the respondents in both groups were unaware of the possible significance of their symptoms. Over as third of respondents (39%) said that they delayed in seeking care because they were 'not concerned' about their symptoms.

**Table 2:** TB knowledge of the migrants and residents in the first round survey

		Migrants %(N)	Residents %(N)	P values
1. Have you heard of TB?	Yes	74.2 (170)	87.1 (676)	P > 0.05
	No	25.8 (59)	12.9 (100)	
2. Does TB have the symptom of chronic cough?	Yes	32.3 (74)	45.1 (350)	P < 0.01
	No	67.7 (155)	54.9 (426)	
3. Does TB have the symptom of hemoptysis?	Yes	36.7 (84)	64.4 (500)	P < 0.01
	No	63.3 (145)	29.1 (226)	
4. Is TB communicable?	Yes	62.9 (144)	82.5 (640)	P < 0.01
	No	37.1 (85)	17.5 (136)	
5. Can TB be treated free?	Yes	38.0 (87)	53.2 (413)	P < 0.01
	No	62.0 (142)	46.8 (363)	
6. Have you heard of TB dispensary?	Yes	61.2 (93)	58.6 (309)	P > 0.05
	No	38.8 (59)	41.4 (218)	

Knowledge and perceptions of the policy of offering free TB diagnosis and treatment for smear positive patients through designated health facilities (largely TB dispensaries) was also influential on treatment seeking behaviour. Although implementation of this policy began in the study areas in 1992, 47% of residents and 62% of migrants knew nothing about it.

The qualitative study findings support the above results and further explored respondents' perceptions of the free TB diagnosis and treatment policy. FGDs with migrants from the general population indicated that the majority had never heard of the policy. Although a minority had heard of it, most of these held some misconceptions about it or did not believe in its existence. For instance, some thought that only those with leukaemia, or those employed in particular sectors could obtain free treatment, whilst others thought that only injections were provided free of charge. Some had heard that not all the services related to TB treatment were free, but did not have any more detailed information. Others were not convinced that the policy was actually being implemented:

*"I do not believe that [there is free treatment]. Even if there are relevant policies developed by the government, perhaps those people at the grass roots will not implement them." (Female casual worker from the FGD)*.

Findings from the in-depth interviews with migrant TB suspects were similar. Very few migrants (3/13) interviewed had heard of the free treatment policy. A greater proportion of residents had heard of the policy (3/7), although few knew any details. In both groups, a few interviewees perceived that it would be difficult to apply for free TB diagnosis and treatment, and some thought that the free TB drugs were poor quality. Some perceptions were partly based on the experiences of acquaintances:

*"When I went home (to the rural area) last time, a village doctor said I might get TB because I coughed for a long... and TB treatment was free... [I felt] there must be much more troubles (to access free treatment)... Someone who visited CDC (Center for Disease Control and Prevention) before told me that he was given many medical tests, some of which was free and others not, and these required many procedures." (Male migrant TB suspect, 55 years old, a factory worker)*.

The perceptions of TB patients were based on their own experiences of trying to access free diagnosis and treatment. The majority of both migrant and resident TB patients expressed dissatisfaction with the free diagnosis and treatment policy. The main reason for this was that some tests and drugs for liver protection prescribed by doctors were not free and they were much more expensive than the cost of anti-TB drugs. As a result, several resident patients even considered the free diagnosis and treatment policy to be a lie:

*"'Free treatment' – I think it is just a lie... Only the sputum smear test was free, and I paid for X-ray examination, blood test and liver function test. I am taking some drugs for liver protection that I also pay for. It's not free."(Male resident patient, a laid-off worker)*.

### Motivation to contact a health care delivery point

This step includes how quickly TB suspects seek care and at which type of facility. The first round survey found that 68% of the migrants delayed seeking care for more than 2 weeks after symptoms appeared (median 23 days), compared to 54% of the residents (median 18 days) (p < 0.01). Before they sought professional care, 80% of migrants and 88% of residents took self-treatment (p < 0.01). Table 3 presents the type of facilities utilized by migrants and residents prior to the first and follow-up surveys. When they decided to seek care at a health facility, over 50% from each group selected district and municipal hospitals as their first service providers. More than 37% of the migrants selected private clinics or 'community clinics' (run by public health facilities but offering similar services, essentially focusing on primary care,) which is 1.5 times as high as the residents. However, almost no patients selected the TB dispensary as their first contact point.

**Table 3:** Pattern of service use between the migrants and the residents in the two survey rounds

	The first patient survey			The follow-up patient survey		
	
Health facility	Migrants % (N)	Residents % (N)	Total % (N)	Migrants % (N)	Residents % (N)	Total % (N)
Private clinic/Community clinic	37.1 (85)	24.4(189)	27.2 (274)	20.0 (5)	11.3(24)	12.2 (29)
District hospital	44.1 (101)	50.6(393)	49.2 (494)	40.0(10)	56.3(120)	54.6(130)
Municipal hospital	14.0 (32)	13.4(104)	13.5 (136)	24.0 (6)	17.3 (37)	18.1 (43)
TB dispensary	0	0.4 (3)	0.3 (3)	12.0(3)	1.0 (2)	2.1 (5)
Other hospitals	4.8 (11)	11.2 (87)	9.8 (98)	4.0(1)	14.1 (30)	13.0(31)

**Figure 2:**
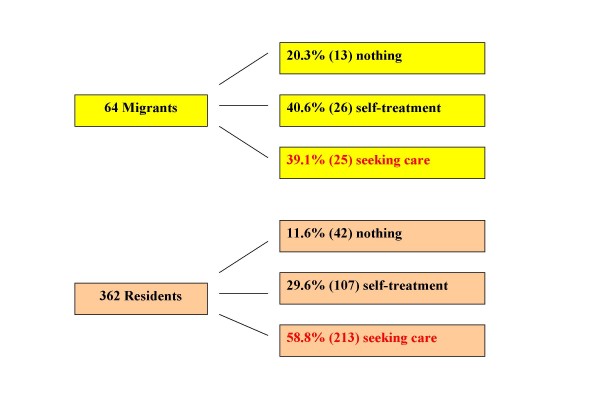
**Comparison of health seeking behaviour among migrants and residents who were still suffering from the symptoms in the follow-up survey**. The chart compares the measures taken in the follow-up survey among the migrants and the residents who were still suffering from the symptoms.

In the follow up survey, 61% of the migrants and 41% of the residents who still had symptoms gave up continuing to seek professional care (Figure 2). The difference between the two groups is statistically significant (p < 0.05). Among those who sought professional care, more patients selected the district and municipal hospital as well as TB dispensary, and fewer selected private clinics/community clinics.

The in-depth interviews with TB suspects indicated similar pathways to care. The majority of migrant TB suspects initially took no measures when cough symptoms began, but when the symptoms lasted from between several days to two months, the majority of migrants (9/13) visited pharmacies to buy some medicine because they felt this was a convenient and cheap option. If the symptoms continued or worsened after this, the majority sought care at health facilities. Most chose private clinics or publicly-run community clinics (8 cases) and a minority went to the enterprise-run hospital, district hospital, or Chinese traditional hospital (4 cases). Private and community clinics were viewed as a particularly accessible option by migrants due to the low cost of their services. One male migrant decided to go back to his rural home to seek care. The following case history was typical of a migrant respondent:

*"At the beginning, I thought it might just be a cold, so after three or four days, I went twice to a pharmacy to buy some drugs. But I felt I was coughing more heavily after taking the medicine, so I decided to go to see a doctor in a private clinic." (Female migrant TB suspect, 30 years old)*.

The resident TB suspects had a similar pathway to care, but tended to wait shorter time intervals between the onset of symptoms and visiting pharmacies, with the longest being ten days. The first health facilities visited by the majority of residents after the failure of medicine obtained from pharmacies were enterprise-run hospitals and community health centres. Enterprise-run hospitals are often designated health facilities identified by the health insurance scheme for the urban residents:

*"I went to our enterprise-run hospital. Because I am one of the workers of this factory, so I got some free services." (Male resident TB suspect, 50+ years old, retired)*.

### Selection

In our application of the Piot model, selection means that the health professional suspects TB and requests a sputum examination and/or X-ray examination. This is intimately associated with the capacity of health providers, including the knowledge of TB and the TB control programme among staff at general health facilities.

We investigated this capacity and knowledge through qualitative interviews with staff at non-specialist institutions. These interviews found that almost all the health workers from general health facilities at all levels were able to identify potential signs of TB correctly, while only one doctor from a private clinic recognised chronic cough over several years as a symptom suggestive of TB. Informal observation at private clinics and community health centres during the conduct of the study found that there were no X-ray machines available at these clinics. The majority of general health providers did know the report and referral system, namely that doctors should report and refer TB suspects/cases seeking care in general health facilities to the local TB dispensary to confirm the diagnosis and start treatment. Most agreed that TB suspects/cases should be referred to TB dispensaries from lower level or general health facilities. A few doctors thought that high level hospitals needed to report TB cases, but not necessarily to refer cases.

However, the actual practice of general health facility staff was also revealed by the questionnaire survey. Only 19% of the migrant patients and 33% of the resident patients, who visited private clinics/community health centres were referred to higher level health facilities for further diagnostic tests (p < 0.05). By the end of the survey, only 5% of the patients were referred to local TB dispensary. There is no significant difference between the two groups (p > 0.05).

Few of the suspects participating in qualitative interviews were referred to TB dispensaries by doctors in health facilities at all levels. Possible reasons for this situation given by doctors in interviews included: first that the quality of services for diagnosis and treatment of TB was better at general hospitals than at the TB dispensaries; and second, that some TB suspects/cases would be reluctant to be referred to the TB dispensaries because of the fear of stigma. Key informants from the district health bureau also agreed that doctors in general hospitals evaded the regulation to report and refer TB cases, often by reporting new cases as relapsed cases, due to their (erroneous) perception that the policy allows them to treat these patients:

*"Last year, we did a survey in a big general hospital. They did not report and refer any TB patients. But we found that many TB patients were getting treatment there, and all of them were registered as relapse cases. That is unbelievable, because the number of cases had increased compared with previous year, so these patients must have been newly occurring cases!" (Leader of disease prevention, District Health Bureau)*.

In addition to this poor referral practice, interviews with health workers in general health facilities found that the majority know little about the free TB diagnosis and treatment policy. Most of them had heard about this policy, but they were not sure who was covered and what benefit the patients could gain. Most doctors said they did not inform TB suspects/cases about the policy due to their own lack of sufficient information about it. When asked for their opinions about the policy, a majority of health providers thought that it could reduce the patient's financial burden related to TB and would be good for TB control. But others remarked that some extra tests and drugs, such as drugs for liver protection, provided during treatment were much more expensive than the free anti-TB drugs so that poor patients still could not afford treatment.

### Examination Procedures

According to the guideline for TB diagnosis in China, TB suspects should first be provided with chest fluoroscopy. Those with suspicious fluoroscopy findings should have three sputum samples examined by smear microscopy, and X-ray examination performed if indicated. The first round of the questionnaire survey found that providers ordered X-ray examinations for 61% of the patients, while only 19% of the patients were prescribed a sputum smear test. There is no significant difference between the migrants and residents (p > 0.05) (Table 4). In qualitative interviews with doctors, most expressed the view that they could confirm a TB diagnosis with an X-ray and few mentioned sputum tests.

**Table 4:** Relevant diagnostic test among TB suspects in two survey rounds

	X-ray examination		Sputum smear test	
	
	Prescribed by doctor % (N)	Actual take-up % (N)	Prescribed by doctor % (N)	Actual take-up % (N)
First survey	61.0(613)	65.4 (401)	19.1 (192)	26.1 (44)
Follow-up survey	38.7 (92)	91.1 (82)	15.2 (36)	83.3 (30)

In addition, many patients did not take the diagnostic tests following the doctors' advice. Only 65% of patients actually took the X-ray examination, and there was no difference between the two groups (p > 0.05). Uptake of sputum smear tests was much lower. Twenty seven percent of the patients from the resident group took the sputum smear tests, which was almost 2.5 times as high as that of the migrants (10%) (p < 0.05).

The leading reasons for not taking X-ray examination were lack of money in the migrant group (35%) and the perception that it was unnecessary in the resident group (59 %). This difference is statistically significant (p < 0.001). The perception that the test was unnecessary was identified as the most important reason for not taking the sputum smear tests by both migrants and residents, whilst the second most important reason was lack of money (Table 5). In the follow up survey, there was a reduction in the proportion of cases where an examination was prescribed, but an increase in patients' adherence in both groups (p > 0.05).

**Table 5:** Self-reported reasons for failure to take diagnostic tests in the first round survey

	X-ray examination		Sputum smear test	
	
Reasons	Migrants % (N)	Residents % (N)	Migrants % (N)	Residents % (N)
No money	34.7 (17)	28.4 (46)	18.4 (7)	22.7 (25)
Unnecessary	30.6 (15)	59.3 (96)	47.4 (18)	69.1 (76)
No time	28.6 (14)	9.9 (16)	10.5 (4)	0.9 (1)
Others	6.1 (3)	2.5 (4)	23.7 (9)	7.3 (8)
Total	100 (49)	100 (162)	100 (38)	100 (110)

Note: X-ray examination: p < 0.001 (1 migrant case missing); Sputum smear test: p = 0.0091

## Discussion

Our findings show that both "patient/personal" and "system/provider" factors interact to influence diagnostic delays at each step of the pathway to diagnosis according to the Piot model. This section discusses the specific factors that combine to act as barriers to accessing health services to attain a TB diagnosis and begin treatment for rural-to-urban migrants in Chongqing.

### Knowledge and awareness of TB and the TB control programme

Our study clearly shows that the knowledge and awareness of TB among the general public and in TB suspects was relatively poor. A third of TB suspects attributed delays to a lack of concern about their symptoms, and the perception that diagnostic tests were not necessary was a major reason for their low uptake. Migrants had significantly less knowledge about TB, possibly due to their lower education level and lower access to health education due to their marginal status in urban areas. As we have reported elsewhere [20], lack of TB knowledge was significantly associated with diagnostic delay in a regression analysis. Lack of knowledge about TB has been associated with 'patient' delay in other studies internationally [21,22].

In addition, our study found that the 'free treatment' policy of the National TB Control Programme is not widely understood or is negatively perceived by the public, which is likely to be a major reason for the very low utilization of TB dispensaries. This may be attributed partly to inadequate dissemination of the policy among the public and TB suspects, including the failure of general health providers to inform their patients, and partly to the limitations of the policy. The complaints documented in our study about the prescription of additional expensive tests and drugs during TB treatment have been also reported in other areas of China [23,24]. The lack of dissemination and limitations of the free treatment policy may particularly affect migrants because of they often have lower financial access to healthcare as discussed below.

### Socio-economic factors affecting access to TB diagnosis

Our study found that the migrants delayed for longer than residents in seeking care and tended to use less expensive health services at the grassroots level on their pathway to diagnosis. Lack of money was also an important factor influencing the uptake of diagnostic tests, particularly for migrants in the case of X-ray tests. The failure of health providers to explain that free diagnostic tests are available at TB dispensaries is discussed below. The majority of migrants have a low educational level, and tend to work in the informal sector where wages are relatively low and unstable. The expectation that they remit money to sustain their rural households further reduces their capacity to pay out-of-pocket payments for healthcare [25]. In addition, they are excluded from the urban social welfare system and as our sample shows, are less likely than residents to have employment-based health insurance.

Another study in China has identified similar factors affecting utilization of health services by migrants, including poverty, lack of health insurance, low education levels, exacting work schedules, and lack of social support [26]. There is also evidence from studies in China that income and educational levels, occupation and low health insurance coverage affect delays in care seeking and diagnosis for TB [12,13,27,28] and access to healthcare in general [29].

### Incentives and capacity in the system for TB diagnosis in urban China

The current Chinese TB control strategy aims to diagnose and treat TB through the TB dispensary system, which is the focus for capacity building and exclusively implements the free diagnosis and treatment policy. However, as our study shows, most TB suspects initiate their health care seeking in general health facilities. Hence, the TB control system relies on the capacity and of general health providers in identifying TB suspects and/or cases and their willingness to refer them to designated TB control institutions.

Our qualitative interviews with health workers from different levels showed they had sufficient knowledge and awareness of TB symptoms to identify suspected cases. However, too few providers ordered the relevant tests and examinations, for a number of possible reasons. In this study, one third of the TB suspects and in particular the migrant patients sought care at private clinics or community health centres. There are no relevant diagnostic facilities available at this grass-roots level, and doctors from this level appear neither to have adequate knowledge (especially in the case of private facilities) or skills to identify TB suspects, nor do they want to refer any TB suspects to higher level or designated health facilities for further diagnosis because of profit-seeking behaviour (discussed below). A study in rural China found that the lower the level at which care was first sought, the longer the diagnostic delay [12]. In the district and municipal hospitals, most doctors did order X-ray examinations for TB suspects as the first step for diagnosis of TB, but they rarely offered sputum smear test to these patients, because either they deem it unnecessary or there is no such facility in the hospital. Establishing TB diagnosis by sputum smear microscopy is one of the key, internationally accepted standards of TB care [30] and a central point of the DOTS strategy.

The length and outcome of the diagnostic process also depends on patient's adherence to professional advice. This requires clear and effective communication between health staff and their patients, including an understanding of patients' attitudes, subjective norms, and social support [31]. Our study found that the uptake of tests recommended by doctors was quite low, often because patients perceived it as "unnecessary". It may be that doctors fail to properly explain the importance of these examinations to their patients.

The finding that lack of money was also an important reason for low uptake of tests suggests that providers are also failing to inform patients that free diagnostic tests are available at TB dispensaries, and some providers admitted this. This failure is related to the financial disincentive to refer patients created by the market-oriented health reforms in China, which require the majority of health facilities to generate revenue through fee-for-service to support their operation. As a consequence, a bonus system is often used to encourage health providers to generate revenue, whereby health providers' income is directly linked to the number of patients treated, the number of examinations provided and the amount of drugs prescribed. This incentive system provides an important explanation for the reluctance of most general health providers to refer their patients, since they may charge patients for TB diagnosis and treatment. Nationally, only 15% of the TB patients diagnosed at general hospitals were reported to and registered with TB dispensaries [32]. Interviews with health providers suggested that lack of knowledge about the free treatment policy also contributed to their failure to refer.

## Conclusion

Our study has shown that 'patient-' and 'provider-' related factors interact to pose barriers to TB diagnosis for rural-to-urban migrants in Chongqing. These factors include: low awareness and poor knowledge of TB and the TB control programme among the general public and TB suspects; low financial capacity to pay for care and diagnostic tests; and inadequate prescription of diagnostic tests and referral to TB dispensaries by general health providers. Rural-to-urban migrants delay for longer in seeking care for symptoms suggestive of TB and are rendered particularly vulnerable to the limitations of the TB control system due to their low incomes, lack of health insurance and low awareness of TB and the free treatment policy. This finding adds further weight to concerns that migrants are less able to realise their rights to healthcare than permanent urban residents and the resulting untreated TB cases are a significant contributor to ongoing TB transmission in urban China. Urgent action to address barriers to diagnosis in both the health systems and communities is required.

## Competing interests

The authors declare that they have no competing interests.

## Authors' contributions

BS, ST, YW and RT initiated the study concept. RT, ST, and YW were responsible for the study design and coordination of the research project. QL, YL, YY and CT performed the data collection, management and analysis. All authors participated in interpretation of the findings. QL drafted the manuscript. RT, ST and BS revised and commented on the draft and all authors read and approved the final version of the paper. All authors confirm that the manuscript has not been published in any journal and other citable form.

## Pre-publication history

The pre-publication history for this paper can be accessed here:


